# Supplementary material for: HSP70 Multi-Functionality in Cancer
Source: Cells. 2020 Mar 2;9(3):587. doi: 10.3390/cells9030587 (PMC7140411; doi:10.3390/cells9030587)
Supplement: Supplementary file 1 [file cells-09-00587-s001.pdf]

Supplementary Materials

HSP70 multi-functionality in cancer

Zarema Albakova[1,2]*, Grigoriy A. Armeev[1], Leonid M. Kanevskiy[2], Elena I. Kovalenko[2] and Alexander M. Sapozhnikov[1,2]

1 Department of Biology, Lomonosov Moscow State University, 119192, Moscow, Russia; armeev@intbio.org (G.A.A); amsap@mail.ru (A.M.S.)

2 Department of Immunology, Shemyakin and Ovchinnikov Institute of Bioorganic Chemistry of the Russian Academy of Sciences; 117997, Moscow, Russia; leonid_kanewski@mail.ru (L.M.K.); lenkovalen@mail.ru (E.I.K.)

* Correspondence: zarema.albakova14@gmail.com

Supplementary figure

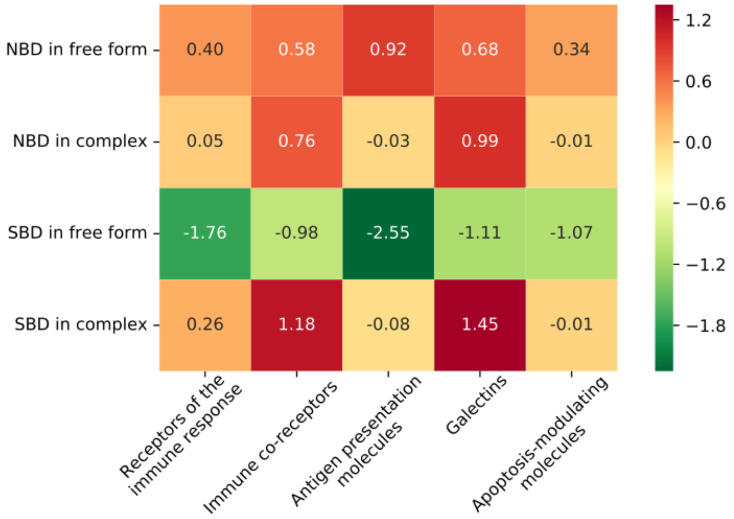

Figure 1. Average Z score of HSP70 interactions. Average Z scores of interaction energies for complexes formed between HSP70-SBD and HSP70-NBD domains in bound and free forms with different classes of interacting molecules.

Supplementary Table

Table 1. Molecules used for protein-protein docking

Classification		PDB ID	Chains selected for docking
SBD in complex		4PO2 [1]	A
		6JPV [2]	A
SBD in free form		5XI9 [3]	A
NBD in complex		2E8A [4]	A
		3ATU [5]	A
		5AQZ [6]	A
NBD in free form		2E88 [4]	A
Receptors of the immune response	Toll-like receptors	TLR1: 6NIH [7]	A
		TLR2: 6NIG [7]	A
		TLR4: 3FXI [8]	A
	NK cell receptors	NKG2A: 3BDW [9]	A
		NKG2D: 1MPU [10]	A
	TCR receptors	TCR $\alpha\beta$: 6JXR [11]	D E F G M N
		TCR$\gamma\delta$: 6MWR [12]	C D
	Nod-like receptors	NLRP3: 6NPY [13]	A
	Complement receptors	CD46: 3INB[14]	C
		CD46: 5FO8[15]	C
Immune co-receptors		CD80: 1DR9[16]	A
		CD86:1NCN [17]	A
		CD40: 3QD6 [18]	S
		CD40: 6PE9 [19]	G
		CD28: 1YJD [20]	C
		PD-L1: 6RPG [21]	A

	CD4: 1CDJ [22]	A
	CD8: 1CD8 [23]	A
Antigen presentation molecules	MHC I: 1HHJ [24]	A
	MHC II: 4I5B [25]	A B
	MHC II: 2IAN [26]	A B
Galectins	Galectin-1: 1GZW[27]	A
	Galectin-9: 3LSD [28]	A
	Galectin-9:3NV1 [29]	A
Apoptosis-modulating molecules	Bcl-2: 2W3L [30]	A
	Apoptosome: 5JUY[31]	B I

Supplementary References

1. Zhang, P., et al., *Crystal Structure of the Stress-Inducible Human Heat Shock Protein 70 Substrate-Binding Domain in Complex with Peptide Substrate.* PLOS ONE, 2014. **9**(7): p. e103518.
2. Lim, S., et al., *Targeting the interaction of AIMP2-DX2 with HSP70 suppresses cancer development.* Nat Chem Biol, 2019.
3. Umehara, K., et al., *Substrate Binding Switches the Conformation at the Lynchpin Site in the Substrate-Binding Domain of Human Hsp70 to Enable Allosteric Interdomain Communication.* Molecules, 2018. **23**(3).
4. Shida, M., et al., *Direct inter-subdomain interactions switch between the closed and open forms of the Hsp70 nucleotide-binding domain in the nucleotide-free state.* Acta Crystallogr D Biol Crystallogr, 2010. **66**(Pt 3): p. 223-32.
5. Arakawa, A., et al., *Biochemical and structural studies on the high affinity of Hsp70 for ADP.* Protein Sci, 2011. **20**(8): p. 1367-79.
6. Cheeseman, M.D., et al., *Exploiting Protein Conformational Change to Optimize Adenosine-Derived Inhibitors of HSP70.* J Med Chem, 2016. **59**(10): p. 4625-36.
7. Su, L., et al., *Structural Basis of TLR2/TLR1 Activation by the Synthetic Agonist Diprovocim.* J Med Chem, 2019. **62**(6): p. 2938-2949.
8. Park, B.S., et al., *The structural basis of lipopolysaccharide recognition by the TLR4-MD-2 complex.* Nature, 2009. **458**(7242): p. 1191-5.
9. Sullivan, L.C., et al., *The heterodimeric assembly of the CD94-NKG2 receptor family and implications for human leukocyte antigen-E recognition.* Immunity, 2007. **27**(6): p. 900-11.
10. McFarland, B.J., et al., *Symmetry recognizing asymmetry: analysis of the interactions between the C-type lectin-like immunoreceptor NKG2D and MHC class I-like ligands.* Structure, 2003. **11**(4): p. 411-22.
11. Dong, et al., *Structural basis of assembly of the human T cell receptor-CD3 complex.* Nature, 2019. **573**(7775): p. 546-552.
12. Le Nours, J.R., J., *Recognition of MHC-like molecule.* 2019.
13. Sharif, H., et al., *Structural mechanism for NEK7-licensed activation of NLRP3 inflammasome.* Nature, 2019. **570**(7761): p. 338-343.

14. Santiago, C., et al., *Structure of the measles virus hemagglutinin bound to the CD46 receptor.* Nat Struct Mol Biol, 2010. **17**(1): p. 124-9.
15. Forneris, F., et al., *Regulators of complement activity mediate inhibitory mechanisms through a common C3b-binding mode.* Embo j, 2016. **35**(10): p. 1133-49.
16. Ikemizu, S., et al., *Structure and dimerization of a soluble form of B7-1.* Immunity, 2000. **12**(1): p. 51-60.
17. Zhang, X., et al., *Crystal structure of the receptor-binding domain of human B7-2: insights into organization and signaling.* Proc Natl Acad Sci U S A, 2003. **100**(5): p. 2586-91.
18. An, H.J., et al., *Crystallographic and mutational analysis of the CD40-CD154 complex and its implications for receptor activation.* J Biol Chem, 2011. **286**(13): p. 11226-35.
19. Argiriadi, M.A., et al., *CD40/anti-CD40 antibody complexes which illustrate agonist and antagonist structural switches.* BMC Mol Cell Biol, 2019. **20**(1): p. 29.
20. Evans, E.J., et al., *Crystal structure of a soluble CD28-Fab complex.* Nat Immunol, 2005. **6**(3): p. 271-9.
21. Basu, S., et al., *Design, Synthesis, Evaluation, and Structural Studies of C2-Symmetric Small Molecule Inhibitors of Programmed Cell Death-1/Programmed Death-Ligand 1 Protein-Protein Interaction.* J Med Chem, 2019.
22. Wu, H., et al., *Kinetic and structural analysis of mutant CD4 receptors that are defective in HIV gp120 binding.* Proc Natl Acad Sci U S A, 1996. **93**(26): p. 15030-5.
23. Leahy, D.J., R. Axel, and W.A. Hendrickson, *Crystal structure of a soluble form of the human T cell coreceptor CD8 at 2.6 A resolution.* Cell, 1992. **68**(6): p. 1145-62.
24. Madden, D.R., D.N. Garboczi, and D.C. Wiley, *The antigenic identity of peptide-MHC complexes: a comparison of the conformations of five viral peptides presented by HLA-A2.* Cell, 1993. **75**(4): p. 693-708.
25. Schulze, M.S., et al., *Disruption of hydrogen bonds between major histocompatibility complex class II and the peptide N-terminus is not sufficient to form a human leukocyte antigen-DM receptive state of major histocompatibility complex class II.* PLoS One, 2013. **8**(7): p. e69228.
26. Deng, L., et al., *Structural basis for the recognition of mutant self by a tumor-specific, MHC class II-restricted T cell receptor.* Nat Immunol, 2007. **8**(4): p. 398-408.
27. Lopez-Lucendo, M.F., et al., *Growth-regulatory human galectin-1: crystallographic characterisation of the structural changes induced by single-site mutations and their impact on the thermodynamics of ligand binding.* J Mol Biol, 2004. **343**(4): p. 957-70.
28. Solis, D., et al., *N-domain of human adhesion/growth-regulatory galectin-9: preference for distinct conformers and non-sialylated N-glycans and detection of ligand-induced structural changes in crystal and solution.* Int J Biochem Cell Biol, 2010. **42**(6): p. 1019-29.
29. Yoshida, H., et al., *X-ray structures of human galectin-9 C-terminal domain in complexes with a biantennary oligosaccharide and sialyllactose.* J Biol Chem, 2010. **285**(47): p. 36969-76.
30. Porter, J., et al., *Tetrahydroisoquinoline amide substituted phenyl pyrazoles as selective Bcl-2 inhibitors.* Bioorg Med Chem Lett, 2009. **19**(1): p. 230-3.
31. Cheng, T.C., et al., *A near atomic structure of the active human apoptosome.* Elife, 2016. **5**.
